# Preoperative C-reactive protein to albumin ratio and oral health in oral squamous cell carcinoma patients

**DOI:** 10.1186/s12903-021-01516-0

**Published:** 2021-03-19

**Authors:** Arvi Keinänen, Johanna Uittamo, Magdalena Marinescu-Gava, Satu Kainulainen, Johanna Snäll

**Affiliations:** 1 Department of Oral and Maxillofacial Diseases, University of Helsinki and Helsinki University Hospital, P.O. Box 220, 00029 Helsinki, Finland; 2 HUS Radiology (Medical Imaging Center), Helsinki, Finland; 3 Finnish Student Health Service, Helsinki, Finland

**Keywords:** Oral cancer, CRP/albumin ratio, Oral health, Cancer survival

## Abstract

**Background:**

The C-reactive protein to albumin (CRP/alb) ratio can predict early survival of a hospitalized patient. We evaluated factors that influence the preoperative CRP/alb ratio in oral squamous cell carcinoma (OSCC) patients and in particular clarified the role of oral health to this ratio.

**Materials and methods:**

Data from surgically treated OSCC patients were collected retrospectively. The outcome variables were preoperative CRP/alb ratio, CRP level, and alb level. The studied predictors were total number of teeth, periodontal stability, marginal bone loss, tumour stage, T-class, lymph node status, and site. The statistical significance of age, sex, comorbidity combination of age and disease history (Charlson Comorbidity Index [CCI]), smoking, and alcohol history for outcome variables were evaluated. Patient 3-month mortality and occurrence of postoperative infections were recorded.

**Results:**

A total of 159 patients were included in the study. The early mortality was 3.8%. CRP/alb was higher in these patients than in those who survived. The only independent variables for CRP/alb changes were CCI and heavy alcohol use. The CRP/alb ratio was significantly lower in non-heavy alcohol users (odds ratio [OR] 0.114, 95% confidence interval [CI] 0.024–0.541; adjusted *p* = 0.006) than in other patients. Patients with CCI 0–1 were more likely to have a lower CRP/alb ratio than patients with CCI ≥ 5 (OR 0.033, 95% CI 0.004–0.284; adjusted *p* = 0.002). In addition, high CRP/alb ratio associated with postoperative infections (*p* = 0.026).

**Conclusions:**

The CRP/alb ratio was high in OSCC patients with combined comorbities of age and disease history and in patients with heavy alcohol use. Oral health or tumour-related variables did not independently affect the CRP/alb ratio. The CRP/alb ratio appears suitable for prediction of OSCC patient early survival.

## Background

The predominant type of oral cancer is oral squamous cell carcinoma (OSCC), which comprises 90% of all oral carcinomas [1]. The main risk factors for the development of OSCC are alcohol consumption and tobacco smoking [2]. The survival rate of patients with OSCC depends on many factors, such as age, gender, diagnosis time, socioeconomic background, and tumour, node, and metastasis (TNM) classification [3]. In particular, the OSCC survival rate is highly dependent on cancer staging [4]. Disease-specific survival beyond 3 years is approximately 70% in patients with stage-I tumours, whereas in stage III/IV OSCCs only 60 % patients survive beyond 3 years [4].

It has been suggested that in a malignant tumour, inflammatory cells act as early intrinsic defence mechanisms to resist the tumour. However, chronic inflammation results in tumour angiogenesis and DNA damage, which both increase C-reactive protein (CRP) levels [5, 6]. OSCC patients with larger tumours and more advanced tumour stages have reduced overall survival when increased CRP levels are present [7, 8]. Thus, the CRP level is considered to increase the prognostic power in OSCC patients.

In addition to CRP, the chronic phase protein albumin (alb) is an indicator of the patient’s nutritional and inflammatory status [9, 10]. Systemic inflammation, followed by a decrease in alb level, results in poor performance, weight loss, and nutritional deficiency, all of which negatively affect the prognosis of patients with cancer [11]. Serum alb levels are lower in patients with oral pre-malignancy and oral malignancy compared with healthy individuals. In addition, increased salivary alb levels have been observed in patients with oral pre-malignancy and oral malignancy compared with healthy individuals [12].

CRP is an acute-phase infective and inflammatory process protein. An increase in CRP production and diminished alb as a negative acute-phase protein are most often associated with chronic disease [13, 14]. The ratio of CRP/alb correlates particularly well with the modified early warning score (MEWS), which is associated with a poorer outcome in many systemic diseases [14]. Fairclough et al., 2009 observed in a follow-up study of 300 patients that the CRP/alb ratio was generally better than MEWS as a predictor of death in younger patients [14]. The CRP/alb ratio is a novel inflammation-based prognostic score and has shown outstanding prognostic value in tumours such as those of the liver or lung [15–17]. The CRP/alb ratio is comparable or even superior to other inflammation-based prognostic scores in predicting prognosis [15–17].

Oral health and particularly the infectious load of periodontitis may have systemic effects [18]. There are some studies on the associations between oral health and serum alb levels [18–20]. The role of oral microbes is associated with the inflammatory process itself and cancer risk [21], and the relationship between periodontal status and cancer prognosis has been evaluated. The mortality of orodigestive cancers increases according to the severity of periodontitis [22]. Additionally, the periodontal pathogen *Porphyromonas gingivalis* has been presented as a biomarker for microbe-associated risk of death [22].

The purpose of the present study was to evaluate the factors that influence preoperative CRP and alb levels and particularly the CRP/alb ratio in patients with OSCC. We also focused on the role of oral health. We hypothesized that the CRP/alb ratio differs between OSCC patients.

## Materials and methods

### Patient material

Patient records from the 3-year period between January 2016 and December 2018 at the Head and Neck Centre, Helsinki University Hospital, Helsinki, Finland were evaluated. Our search was based on data from the multidisciplinary head and neck tumour board of Helsinki University Hospital, which covers data of all primary diagnosed OSCC patients.

Data on OSCC patients and tumours, laboratory values, radiological dental status, information on smoking and alcohol consumption habits, and early overall survival were collected retrospectively.

### Inclusion and exclusion criteria

Patients with a primary OSCC diagnosis who received surgical treatment for the malignancy were included. An additional inclusion criterion was the availability of a digital panoramic radiography (DPR) of dentate patients. Patients with additional malignancy, previous head or neck malignancy, previous radiotherapy in the region of head or neck, preoperative acute infection, and missing preoperative CRP or alb values or incomplete information on smoking or alcohol consumption were excluded.

### Study design

The primary outcome variable was CRP/alb ratio before treatment. Additional outcome variables were CRP level (mg/l) and alb (g/l) levels before treatment. The lowest value of CRP that could be obtained by laboratory tests was < 3 mg/l.

Predictor variables related to oral health were periodontal stability, marginal bone loss, and number of teeth. Patients were grouped according to number of teeth, which included third molars and excluded dental implants. In assessing periodontal status, unerupted teeth, teeth without sound supragingival tissue remaining (crown affected by caries), and teeth floating in parts of the jaw affected by the tumour were excluded.

Tumour-related predictor variables included prognostic stage group (i.e. cancer severity), tumour size, patient lymph node status, and tumour site. The prognostic stage group was defined according to the Oral Cavity cancers—AJCC 8th Edition into two subgroups (stage 0-II and stage III-IV) [23]. Correspondingly, tumour size was defined according to T categorization as Tis-2 (Tis, T1, or T2) or T3-4 (T3 or T4). Pathological lymph node status was categorized as N0 and N1 or more.

Explanatory variables were age, gender, smoking, heavy alcohol use, and Charlson Comorbidity Index (CCI) [24], which combines comorbidity for age and disease history. Patients were divided into three CCI groups as follows: 0–1 points, 2–4 points, and ≥ 5 points [24]. Patients were stratified by smoking habit into the following two groups: non-smokers (i.e. non-smokers and former smokers who have been in cessation ≥ 5 years) and current and former smokers (i.e. who have been in cessation < 5 years) [25]. Alcohol use was determined according to the Finnish Current Care Guidelines consumption limits for heavy alcohol use: ≥ 23 doses (i.e. ≥ 287.5 g of alcohol) per week for men and ≥ 12 doses (i.e. ≥ 150 g alcohol) per week for women as suggested by Finnish working group [26].

Patients early mortality and occurrence of postoperative infections during the first three months were recorded. Associations between early mortality and postoperative infections and the CRP/alb ratio were reported.

### Radiological analyses for periodontal status

Dental panoramic radiograph images (Instrumentarium Dental™ Orthopantomograph™ OP200 or Orthopantomograph® OP300) were reviewed by an oral radiologist (M.M.G.) twice at 6-month intervals. For discordant results, the more severe status was used for the study.

**Fig. 1:**
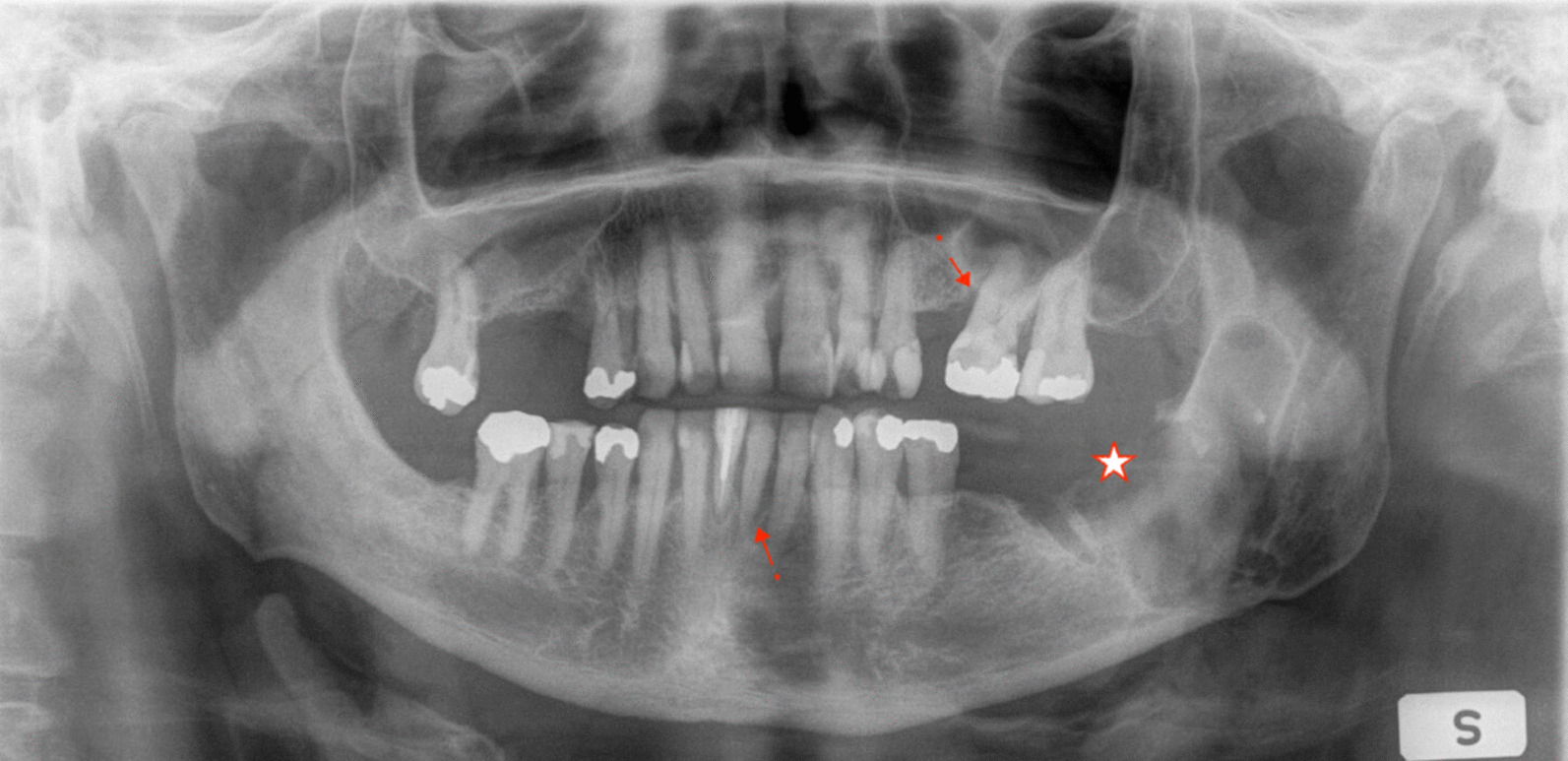
Panoramic tomography of a patient with missing teeth and periodontal instability, with severe marginal bone loss (up to apical third of the roots, marked with *). On the left there is also tumour-induced bone destruction (star), which in this case does not involve a dentate area of the mandible.

Periodontal status was evaluated for periodontal stability and marginal bone loss. Stability was defined according to alveolar marginal crest and described as stable if a corticated alveolar marginal crest was radiologically identifiable (i.e. presence of a corticated crestal lamina dura). When present, marginal bone loss was classified as not present, mild, moderate, or severe [27]. Marginal bone loss was determined by tertile as follows: maximum bone loss extending to cervical third (mild), maximum bone loss extending to the middle third (moderate), or maximum bone loss extending to the apical third (severe) (Fig. 1). Edentulous patients were included as patients with periodontal stability without marginal bone loss.

### Statistical analysis

Associations between patient cancer severity status, clinical data, periodontal status, early death, postoperative infection and CRP, alb and CRP/alb ratio were assessed with the Chi-squared test, Fisher’s exact test, and logistic regression analysis based on median values of the population. Hosmer and Lemeshow goodness-of-fit test was used to assess logistic regression analyses. Before conducting multiple logistic regression analyses, Cramer’s V test was used to detect possible multicollinearity of categorical explanatory variables. The significance level was set at 0.05. SPSS 25.0 (IBM Corp, Armonk, NY) was used for statistical analyses.

### Ethical approval

The study was approved by the Internal Review Board of the Head and Neck Centre, Helsinki University Central Hospital, Finland (HUS/66/2018).

## Results

### Patient material

**Fig. 2:**
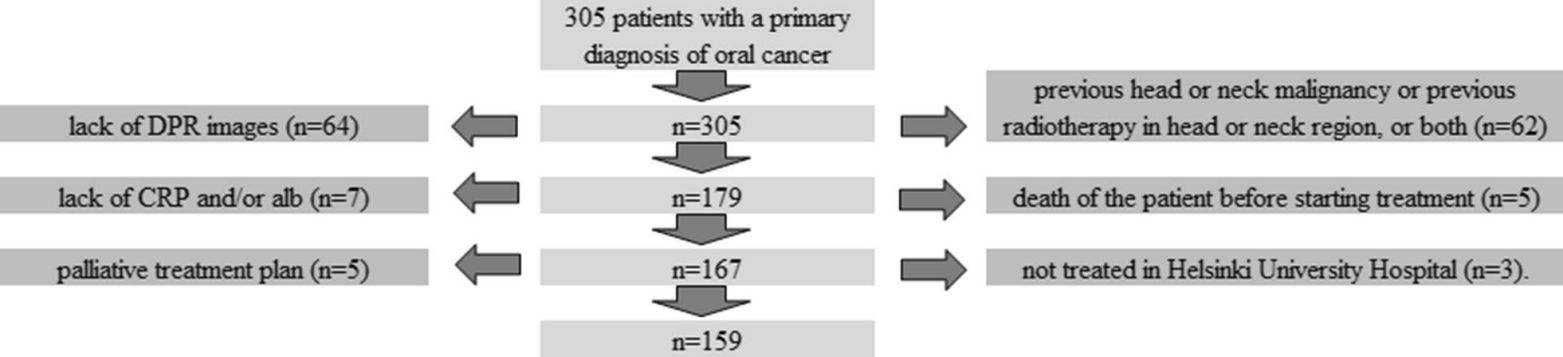
In all remaining 159 patients, requisite data on smoking and alcohol consumption were available. Thus, 159 patients were included in the final analyses.

**Fig. 3:**
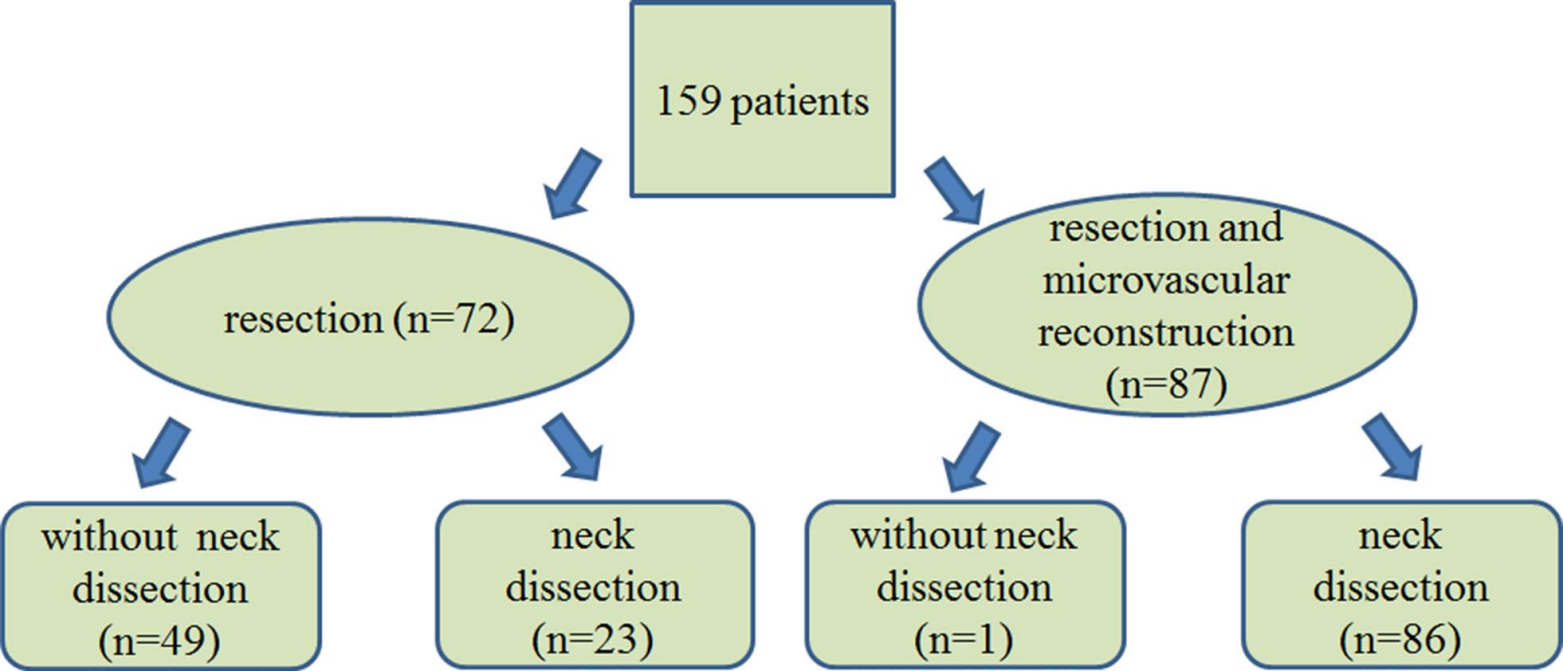
All 159 oral squamous cell carcinoma patients underwent tumour resection.

From a total of 305 evaluated patients, 159 OSCC patients who received surgical treatment for primary tumour were included in the final analyses (Fig. 2). In all, 87 patients received microvascular reconstruction in addition to tumour resection (Fig. 3) and 99 patients were given additional chemoradiotherapy. All patients received perioperative antibiotic medication, which was continued as needed postoperatively according to the type of procedure. Most patients were male (51.6%). Median age was 66.0 years. Most patients were non-smokers (50.3%) and did not have a history of heavy alcohol use (82.4%). The most common primary tumour site was tongue (44.0%), followed by gingiva or palate (34.0%), floor of the mouth (15.7%), and cheek (6.3%). Median values for the studied laboratory variables were the following: CRP < 3 mg/l, alb 38.00 g/l, and CRP/alb 0.05 (mg/l)/(g/l) (Table 1).

**Table 1:** Descriptive statistics of 159 oral carcinoma patients.

		No. of patients	%	♂/♀	Gender		*p* value
					Male (%)	Female (%)	
*Age (years)*							
Range	23–96						
Mean	66.83						
Median	66.00						
< 66		77	48.4	48/29	58.5	37.7	
≥ 66		82	51.6	34/48	41.5	62.3	**0.011**
*Gender*							
Male		82	51.6				
Female		77	48.4				
*Smoking*							
Non-smoker		80	50.3	36/44	43.9	57.1	
Current Smoker		79	49.7	46/33	56.1	42.9	0.113
*Heavy alcohol use*							
No		131	82.4	65/66	79.3	85.7	
Yes		28	17.6	17/11	20.7	14.3	0.306
*Site*							
Tongue		70	44.0	35/35	42.7	45.5	
Gum or palate		54	34.0	28/26	34.1	33.8	
Floor of the mouth		25	15.7	15/10	18.3	13.0	
Cheek		10	6.3	4/6	4.9	7.8	0.734
*Periodontal stability*							
Present		77	48.4	32/45	39.0	58.4	
Not present		82	51.6	50/32	50.0	41.6	**0.017**
*Marginal bone loss*							
Not present		23	14.6	11/12	13.6	15.6	
Mild		58	36.7	29/29	35.8	37.7	
Moderate		57	36.1	28/29	34.6	37.7	
Severe		20	12.7	13/7	16.0	9.1	0.636
*Total number of teeth*							
Range	0–32						
Mean	19.73						
Median	23.00						
0		16	10.1	7/9	8.5	11.7	
1–12		16	10.1	7/9	8.5	11.7	
13–24		64	40.3	32/32	39.0	41.6	
25–32		63	39.6	36/27	43.9	35.1	0.655
*Charlson Comorbidity Index*							
0–1		32	20.1	21/11	25.6	14.3	
2–4		106	66.7	53/53	64.6	68.8	
≥ 5		21	13.2	8/13	9.8	16.9	0.130

Statistically significant values are in bold

In all, 6 patients died during the first postoperative three months (3.8%). The cause of death was cardiovascular in 3 patients, respiratory infection or respiratory complication in 2 patients and 1 patient died while receiving palliative cancer care. Of the deceased patients, three had T4, two had T3 and one had T2 tumour. Four of these six patients received microvascular reconstruction. The CRP/alb ratio was higher in these patients compared to surviving patients (Table 2).

**Table 2:** Early postoperative mortality, postoperative infections and CRP/alb ratio in oral squamous cell carcinoma patients

	Min	Max	Mean	Median	CRP/alb				
					≤ 0.05		0.05<		*p* value
					n (of 41)	%	n (of 118)	%	
*Early death*									
No (n = 153)	0.04	1.73	0.16	0.05	40	26.1	113	73.9	
Yes (n = 6)	0.05	0.97	0.32	0.19	1	16.7	5	83.3	>0.999
*Postoperative infection*									
No (n = 134)	0.04	1.73	0.14	0.05	39	29.1	95	70.9	
Yes (n = 25)	0.05	1.71	0.31	0.09	2	8.0	23	92.0	**0.027**

Statistically significant values are in bold

In all, 25 of 159 patients (15.7%) had infections during the first postoperative three months. In eleven patients infections were associated with surgical site, nine patients had pneumonia, two had urinary tract infections, one had salivary gland infection, one had infection associated with tracheostomy and one had infection associated with a gastrostomy tube. Patients with higher CRP/alb ratio were significantly more likely to have postoperative infections than patients with lower CRP/alb ratio (*p* = 0.026) (Table 2).

Smokers had significantly higher CRP levels (*p* = 0.036) and higher alb levels (*p* = 0.026) than non-smokers (Table 3). Patients with a history of heavy alcohol use had significantly higher CRP levels (*p* = 0.001) and a higher CRP/alb ratio (*p* = 0.016) compared with the other patients. Patients with a higher CCI score more often had lower alb levels (*p* < 0.001) and a higher CRP/alb ratio (*p* < 0.001). Associations between explanatory variables and CRP/alb ratio are shown in Table 4.

**Table 3:** Association between the laboratory tests and patient data in 159 oral carcinoma patients

	CRP					alb				
	< 3		3≤			≤ 38		38<		
	n (of 96)	%	n (of 63)	%	*p* value	n (of 83)	%	n (of 76)	%	*p* value
*Age (years)*										
<66	44	57.1	33	42.9		34	44.2	43	55.8	
≥66	52	63.4	30	36.6	0.517	49	59.8	33	40.2	0.057
*Gender*										
Male	48	58.5	34	41.5		41	50.0	41	50.0	
Female	48	62.3	29	37.7	0.631	42	54.5	35	45.5	0.634
*Smoking*										
non-smoker	68.8	25	31.3			49	61.3	31	38.8	
current smoker	51.9	38	48.1	**0.036**		34	43.0	45	57.0	**0.026**
*Heavy alcohol use*										
No	87	66.4	44	33.6		67	51.1	64	48.9	
Yes	9	32.1	19	67.9	**0.001**	16	57.1	12	42.9	0.678
*Charlson Comorbidity Index*										
0–1	23	71.9	9	28.1		7	21.9	25	78.1	
2–4	64	60.4	42	39.6		61	57.5	45	42.5	
≥ 5	9	42.9	12	57.1	0.123	15	71.4	6	28.6	**<0.001**

Statistically significant values are in bold

**Table 4:** Association between the laboratory tests and patient data in 159 oral carcinoma patients

	CRP/alb				
	≤0.05		0.05<		
	n (of 41)	%	n (of 118)	%	*p* value
*Age (years)*					
< 66	25	32.5	52	67.5	
≥ 66	16	19.5	66	80.5	0.071
*Gender*					
Male	23	28.0	59	72.0	
Female	18	23.4	59	76.6	0.587
*Smoking*					
Non-smoker	21.3	63	78.8		
Current smoker	30.4	55	67.6	0.208	
*Heavy alcohol use*					
No	39	29.8	92	70.2	
Yes	2	7.1	26	92.9	**0.016**
*Charlson Comorbidity Index*					
0–1	16	50.0	16	50.0	
2–4	24	22.6	82	77.4	
≥ 5	1	4.8	20	95.2	**<** **0.001**

Statistically significant values are in bold

Primary tumour sites differed significantly when considering alb levels (*p* = 0.027) (Table 5). Patients with floor of the mouth tumours had the highest alb levels compared with other regions. T-class was significantly associated with CRP levels (*p* = 0.030). Patients with larger tumours more often had higher CRP than patients with less extensive tumours. Differences were also significant between total number of teeth and CRP/alb ratio (*p* = 0.009) (Table 6). Edentulous patients and patients with 13–24 teeth more often had a lower CRP/alb ratio than other patients.

**Table 5:** Association between the laboratory tests and predictor variables in 159 oral carcinoma patients.

	CRP					alb				
	< 3		3≤			≤ 38		38<		
	n (of 96)	%	n (of 63)	%	*p* value	n (of 83)	%	n (of 76)	%	*p* Value
*Periodontal stability*										
present	52	67.5	25	32.5		41	53.2	36	46.8	
not present	44	53.7	38	46.3	0.078	42	51.2	40	48.8	0.874
*Marginal bone loss*										
not present	15	65.2	8	34.8		12	52.2	11	47.8	
mild	38	65.5	20	34.5		23	39.7	35	60.3	
moderate	32	56.1	25	43.9		34	59.6	23	40.4	
Severe	10	50.0	10	50.0	0.547	14	70.0	6	30.0	0.060
*Total number of teeth*										
0	9	56.3	7	43.8		10	62.5	6	37.5	
1–12	9	56.3	7	43.8		8	50.0	8	50.0	
13–24	36	56.3	28	43.8		39	60.9	25	39.1	
25–32	42	66.7	21	33.3	0.620	26	41.3	37	58.7	0.129
*Stage*										
I–II	54	65.1	29	34.9		42	50.6	41	49.4	
III–IV	42	55.3	34	44.7	0.256	41	53.9	35	46.1	0.751
*T-class*										
Tis–T2	67	67.0	33	33.0		49	49.0	51	51.0	
T3–T4	29	49.2	30	50.8	**0.030**	34	57.6	25	42.4	0.327
*Lymph node status*										
N0	73	62.9	43	37.1		61	52.6	55	47.4	
N1 or more						22	51.2	21	48.8	1.000
*Site*										
Tongue	45	64.3	25	35.7		38	54.3	32	45.7	
Gum or palate	30	55.6	24	44.4		34	63.0	20	37.0	
Floor of the mouth	15	60.0	10	40.0		7	28.0	18	72.0	
Cheek	6	60.0	4	40.0	0.793	4	40.0	6	60.0	**0.027**

Statistically significant values are in bold

**Table 6:** Association between the laboratory tests and predictor variables in 159 oral carcinoma patients

	CRP/alb				
	≤0.05		0.05<		
	n (of 41)	%	n (of 118)	%	*p* value
*Periodontal stability*					
Present	22	28.6	55	71.4	
Not present	19	23.2	63	76.8	0.472
*Marginal bone loss*					
Not present	7	30.4	16	69.6	
Mild	19	32.8	39	67.2	
Moderate	12	21.1	45	78.9	
Severe	2	10.0	18	90.0	0.166
*Total number of teeth*					
0	3	18.8	13	81.3	
1–12	4	25.0	12	75.0	
13–24	9	14.1	55	85.9	
25–32	25	39.7	38	60.3	**0.009**
*Stage*					
I–II	22	26.5	61	73.5	
III–IV	19	25.0	57	75.0	0.858
*T-class*					
Tis–T2	30	30.0	70	70.0	
T3–T4	11	18.6	48	81.4	0.135
*Lymph node status*					
N0	28	24.1	88	75.9	
N1 or more	13	30.2	30	69.8	0.541
*Site*					
Tongue	20	28.6	50	71.4	
Gum or palate	11	20.4	43	79.6	
Floor of the mouth	9	36.0	16	64.0	
Cheek	1	10.0	9	90.0	0.313

Statistically significant values are in bold

Multiple logistic regression analysis included heavy alcohol use and CCI for CRP/alb ratio (Table 7). The CRP/alb ratio was significantly lower in patients with no history of heavy alcohol use (odds ratio [OR] 0.114, 95% confidence interval [CI] 0.024–0.541; adjusted *p* = 0.006) than in patients with history of heavy alcohol use. In addition, patients with CCI 0–1 were more likely to have lower CRP/alb ratio than patients with CCI ≥ 5 (OR 0.033, 95% CI 0.004–0.284; adjusted *p* = 0.002).

**Table 7:** Logistic Regression analysis of the CRP/alb in 159 oral carcinoma patients

	Univariate				Multiple			
	> 0.05 of CRP/Alb before treatment				> 0.05 of CRP/Alb before treatment			
		95% CI for OR				95% CI for OR		
	OR	Lower	Upper	*p* value	OR	Lower	Upper	*p* value
*Heavy alcohol use* (*yes f.*)								
no heavy alcohol use	0.181	0.041	0.802	**0.024**	0.114	0.024	0.541	**0.006**
*Charlson Comorbidity Index* (≥ 5 *f.*)								
0–1	0.050	0.006	0.418	**0.006**	0.033	0.004	0.284	**0.002**
2–4	0.171	0.022	1.339	0.093	0.143	0.018	1.130	0.065
*Total number of teeth* (0 *f.*)								
1–12	0.692	0.128	3.752	0.670				
13–24	1.410	0.334	5.950	0.640				
25–32	0.351	0.091	1.357	0.129				

Statistically significant values are in bold

## Discussion

We evaluated the factors that influence preoperative CRP and alb levels and the CRP/alb ratio in OSCC patients. We also evaluated the role of oral health. We hypothesized that the CRP/alb ratio differs between OSCC patients, which was confirmed in this study. Although numerous factors were associated with CRP and alb and CRP/alb, heavy alcohol use, high CCI were found to be the only independent variables for CRP/alb increase. In addition, we found statistically significant association between high CRP/alb ratio and postoperative infections.

CRP/alb scores in OSCC patients have been considered as promising predictive markers for patients with OSCC in addition to tumour staging [28]. A previous study on OSCC patients treated with concomitant radio-chemotherapy showed that CRP and alb predicted patient long-term survival [29]. However, even in this selected patient population, only radiotherapy combined with chemotherapy remained significant in multivariate analyses. While early mortality was low in the present population (3.8%), the CRP/alb ratio was higher in these patients than survivors. Thus, the CRP/alb ratio seems to be more appropriate for assessing early prognosis of OSCC patients.

Preoperative high-sensitivity-CRP (hs-CRP) to alb ratio has previously shown to predict independently signs of severe infection in nephrolithotomy [30]. The CRP/alb ratio predicted postoperative infections also in our data, although hs-CRP was not used. As many as 23 of 25 patients with postop infection had a CRP/alb ratio that exceeded the median of our data. Indeed, in addition to predicting early mortality, CRP/alb appears to be promising for predicting the risk of postoperative infections in oral cancer patients. Further studies should be performed in patient groups where surgical and postoperative care are congruent.

Heavy alcohol use and smoking also increased preoperative CRP, as shown previously [31, 32]. Imhof et al. 2001 found that men’s alcohol consumption showed a U-shaped association with mean CRP values. In their study, non-drinkers and heavy drinkers had higher CRP concentrations than moderate drinkers [32]. In this patient population, only 1 out of 28 heavy alcohol users had liver cirrhosis. According to our results, heavy alcohol use is therefore a notable factor that increases CRP levels in OSCC patients. Even if smokers more often had higher CRP than non-smokers, they also had higher alb values. Thus, no effect of smoking was found on the CRP/alb ratio.

Systemic inflammation in cancer patients is considered to relate to prognosis. Various mechanisms are involved in inflammation cascades during OSCC development and progression. Tumour growth, invasion, or both can cause inflammation via necrosis, tumour hypoxia, or local tissue damage [33–35]. Inflammatory cells related to the tumour and tumour cells related to tumorigenic inflammatory cytokines, such as tumour necrosis factor, interleukin (IL) IL-1, IL-6, and vascular endothelial growth factor [33–35] can induce invasion, growth, and tumour metastasis [33–35]. CRP production increases as a response to these stimuli mediators and has been shown worsen inflammatory status and advance progression of the oral cancer [7, 8, 36]. Thus, it is consistent that tumour extent is associated with CRP levels as confirmed in the present study.

Hypoalbuminemia is a prognostic marker of survival in the general population and in many pathological settings, mainly due to malnutrition and inflammation [37, 38]. Our results are consistent with previous findings. Higher CCI was associated with lower alb levels. Smokers in our study had surprisingly higher alb levels compared to non-smokers. The association between low albumin levels and smoking has been thought to indicate the inflammatory response and degree of vascular changes caused by smoking [39], however, albumin levels are also affected by numerous other factors. In the present study, alb levels were likely to be particularly affected by background diseases in a relatively elderly population, due to which the effect of smoking on alb was not observed in our data. However, it should be noted that a single effect of patient age was not found. Somewhat surprisingly, the number of teeth was also not associated with alb levels.

We chose periodontal stability, marginal bone loss, and total number of teeth as measurements of the level of periodontitis and dental health. Patients that had tooth loss had a significantly higher CRP/alb ratio than patients without tooth loss, but the difference was non-significant in multivariate analysis. Thus, even if OSCC patients often require further dental care as shown in the present study, these findings do not affect the CRP/alb ratio.

The frequent findings of periodontitis in our results are worth considering. Stable periodontal status was observed in only half of the patients and marginal bone loss was frequent. Radiotherapy and chemotherapy have an impact on oral health and the oral cavity should be as free of infection as possible before these oncological treatments. In addition, surgical procedures for OSCC often extend to tooth-bearing regions of the oral cavity. Thus, an individual treatment plan by dentists and oral and maxillofacial surgeons is appropriate to reduce local infections and address other considerations during OSCC treatment, and to promote further rehabilitation of occlusion and jaw function. Appropriate oral and dental care should be included in the OSCC treatment plan to improve the patient’s quality of life.

A larger number of patients with additional data from treatment strategies is required to clarify the clinical relevance of the CRP/alb ratio in specific OSCC patient subgroups. In addition, hs-CRP was not recorded, thus the significance of micro infection burden could not be assessed. An additional limitation is that we estimated periodontal status based on radiological findings alone and cariological status or dental-care habits were not analysed.

In summary, even if a number of factors are associated with the CRP/alb ratio in oral cancer patients, only the comorbidity combination of age and disease history and heavy alcohol use increased the CRP/alb ratio in OSCC patients independently. Consideration of these predictive variables are warranted when assessing early postoperative prognosis of a patient with OSCC.

## Data Availability

The datasets used and/or analysed during the current study are available from the corresponding author on reasonable request.

## Abbreviations

alb: albumin
CRP: C-reactive protein
CRP/alb: C-reactive protein to albumin
CCI: Charlson Comorbidity Index
CI: confidence interval
hs-CRP: high-sensitivity-CRP
IL: interleukin
MEWS: modified early warning score
OSCC: oral squamous cell carcinoma
TNM: tumour, node, and metastasis
